# Functional dissection of velvet DNA-binding region in *Aspergillus nidulans* VelB uncovers an essential conserved arginine cluster

**DOI:** 10.3897/imafungus.17.204179

**Published:** 2026-07-14

**Authors:** Wanping Chen, Anna M. Köhler, Mingrong Deng, Honghui Zhu, Gerhard H. Braus

**Affiliations:** 1 Guangdong Microbial Culture Collection Center, Guangdong Provincial Observation and Research Station for Microbial Science of the Ecosystems of Origin for Geographical Indication Agricultural Products, Key Laboratory of Agricultural Microbiomics and Precision Application (MARA), Key Laboratory of Agricultural Microbiome (MARA), State Key Laboratory of Applied Microbiology Southern China, Institute of Microbiology, Guangdong Academy of Sciences, Guangzhou 510070, China Guangdong Microbial Culture Collection Center, Guangdong Provincial Observation and Research Station for Microbial Science of the Ecosystems of Origin for Geographical Indication Agricultural Products, Key Laboratory of Agricultural Microbiomics and Precision Application (MARA), Key Laboratory of Agricultural Microbiome (MARA), State Key Laboratory of Applied Microbiology Southern China, Institute of Microbiology, Guangdong Academy of Sciences Guangzhou China; 2 Department of Molecular Microbiology and Genetics, University of Göttingen, Göttingen 37077, Germany Department of Molecular Microbiology and Genetics, University of Göttingen Göttingen Germany

**Keywords:** *
Aspergillus
*, DNA-binding, fungi, VelB, velvet regulators

## Abstract

Velvet regulators, characterized by a conserved velvet domain, function as central hubs that coordinately govern fungal development, secondary metabolism, stress adaptation, and pathogenicity. The velvet domain is organized into an N-terminal DNA-binding region of approximately 30 amino acids and a C-terminal dimerization region of approximately 100 amino acids. In this study, *Aspergillus nidulans* VelB was used as a paradigm to systematically dissect the velvet DNA-binding region. The three arginine residues R71, R80, and R81 in the N-terminal velvet domain that are indispensable for VelB function were identified through alanine-scanning mutagenesis of 15 conserved residues. Alanine substitutions at these positions caused severe defects in long-term spore viability, sexual development, and secondary metabolism. Further comparative characterization of electrostatic-potential dynamics pre- and post-mutation revealed that the three individual substitutions markedly attenuated local electrostatic potential across the DNA-binding interface. Notably, these mutations drove comprehensive remodeling of the protein’s electrostatic properties, whereby electrostatic perturbations propagated across the entire protein exterior. Analysis of 4,999 velvet-domain sequences across the fungal kingdom revealed extraordinary conservation of these positions: arginine was present at position 71 in 85% of sequences, at position 80 in 91%, and at position 81 in 84%. Cross-kingdom complementation experiments further demonstrated that the wild-type velvet DNA-binding region from *Capsaspora owczarzaki*, a unicellular holozoan lacking the equivalent of R71, failed to rescue the *A. nidulans velB* deletion phenotype, whereas the introduction of arginine at this position conferred substantial functional restoration. These findings establish that a cluster of conserved arginine residues generates the positive electrostatic surface potential required for velvet–DNA interaction and define the molecular basis of DNA recognition by this ancient family of fungal transcription factors.

## Introduction

The coordination of morphological development with secondary metabolism represents a fundamental challenge in the life cycle of filamentous fungi (Calvo et al. 2002). These organisms must precisely orchestrate the timing of developmental transitions—such as the choice between asexual and sexual reproduction—with the production of bioactive secondary metabolites that serve critical functions in ecological competition, stress adaptation, and chemical defense (Bayram and Braus 2012; Liu et al. 2021). At the molecular level, this coordination is governed by a limited set of conserved transcriptional and epigenetic regulatory networks that integrate diverse environmental cues, including light, temperature, and nutrient availability, to direct appropriate cellular responses. Among these, the velvet family of transcriptional and epigenetic regulatory proteins stands out as a central hub that interconnects fungal development, secondary metabolism, stress adaptation, and pathogenicity, thereby functioning as a master orchestrator of fungal physiology across diverse fungal lineages (Chen et al. 2026).

The velvet regulatory system was first described more than half a century ago in the model filamentous fungus *Aspergillus nidulans*, when Käfer (1965) identified a *veA* (velvet A) mutant strain, designated *veA1*, that exhibited a distinctive developmental phenotype (Käfer 1965). Unlike the wild-type strain, which favors asexual sporulation under illumination and sexual development in darkness, the *veA1* mutant displayed a marked shift toward constitutive asexual sporulation and severely impaired sexual development under normally permissive dark conditions, concomitantly losing the ability to produce major secondary metabolites (Kim et al. 2002; Stinnett et al. 2007). The *veA1* mutant colonies presented a characteristic velvet-like appearance due to their abundant asexual spore production, from which the “velvet” nomenclature was derived (Käfer 1965). Molecular characterization later revealed that the *veA1* allele carries a point mutation in the start codon that truncates the N-terminal 36 amino acids of VeA, thereby abolishing its nuclear localization signal and preventing light-regulated nuclear import (Stinnett et al. 2007).

Subsequent studies revealed that velvet proteins are highly conserved across the fungal kingdom, from early-diverging chytrids to advanced basidiomycetes, with homologs even identified beyond the fungal kingdom (Chen et al. 2024). The founding velvet family encompasses four core members: VeA, VelB (velvet-like B), VelC (velvet-like C), and VosA (viability of spores A) (Kim et al. 2002; Ni and Yu 2007; Park et al. 2012; Park et al. 2014). These proteins form multiple homomeric or heteromeric complexes to implement a diverse range of functional roles, among which the trimeric velvet complex VelB–VeA–LaeA is a prominent example (Bayram et al. 2008; Strohdiek et al. 2025; Köhler et al. 2026). In darkness, VeA translocates into the nucleus and bridges VelB with the methyltransferase LaeA, thereby coordinating sexual development and secondary metabolite production. Under illumination, nuclear localization of VeA is reduced, resulting in altered complex assembly and a shift toward asexual development. This light-responsive regulatory mechanism represents one of the most important paradigms in fungal environmental adaptation.

At the structural level, all velvet proteins share a conserved velvet domain of approximately 200 amino acid residues (Chen et al. 2024). Crystallographic and molecular studies revealed that this domain exhibits structural similarity to the Rel homology domain of mammalian NF-κB transcription factors (Ahmed et al. 2013). A recent study proposed a general architecture of velvet domains consisting of an approximately 30-amino-acid N-terminal DNA-binding region and an approximately 100-amino-acid C-terminal dimerization region that contains α- and β-subunits separated by a flexible linker (Chen et al. 2025). These discoveries significantly advanced understanding of how velvet domains function.

Functional characterization of the C-terminal dimerization region has been established in prior work (Chen et al. 2025), yet the velvet DNA-binding region remains largely uncharacterized. To address this gap, the present study systematically dissected the sequence signatures and core functional residues of the velvet DNA-binding region using *A. nidulans* VelB as a model paradigm. Three key positively charged arginine residues were identified because of their indispensable roles in VelB function and as prerequisites for creating a positive electrostatic surface potential that facilitates interactions between velvet proteins and DNA.

## Materials and methods

### *A. nidulans* and *Escherichia coli* strains and growth conditions

All *A. nidulans* strains used and constructed in this work are summarized in Table 1. Strain AGB551 (*veA^+^*) served as the wild-type reference. Wild-type and mutant strains were cultured in minimal medium (MM). The medium consisted of 1% glucose, 7 mM KCl, 2 mM MgSO_4_, 70 mM NaNO_3_, 11.2 mM KH_2_PO_4_, and 0.1% trace element solution, with the pH adjusted to 5.5, supplemented with 0.1% pyridoxine-HCl, 5 mM uridine, and 5 mM uracil (Barratt et al. 1965; Thieme et al. 2018). Strains were grown for 3 days on solid MM containing 2% agar at 37°C under constant illumination. Conidia were obtained by suspending spores from plate surfaces in sterile 0.96% NaCl solution containing 0.002% Tween 80, followed by removal of hyphal debris through Miracloth filtration. For phenotypic assessment of asexual development, approximately 200 spores were spotted onto the center of MM solid plates and incubated at 37°C under continuous light. For sexual development induction, plates were inoculated similarly, wrapped with Parafilm to prevent gas exchange, and incubated in complete darkness at 37°C.

**Table 1.** *A. nidulans* strains used in this study.

**Strain name**	**Genotype**	**Reference**
AGB551	*∆nkuA::argB*, *pyrG89*, *pyroA4*, *veA^+^*	Bayram et al. 2012
AGB1064	*∆nkuA::argB*, *pyroA4*, *pyrG89*, *veA^+^*, *∆velB::six*	Thieme et al. 2018
AGB1192	*∆nkuA::argB*, *pyrG89*, *pyroA4*, *veA^+^*, *∆velB::velB:sgfp:six*	Thieme 2018
AGB1479	*∆nkuA::argB*, *pyrG89*, *pyroA4*, *veA^+^*, *∆velB::velBcDNA:sgfp:six*	Chen et al. 2025
AGB1481	*∆nkuA::argB*, *pyrG89*, *pyroA4*, *veA^+^*, *velBcDNA(∆velB_DBR::CapVelvet_DBR):sgfp:six*	This study
AGB1484	*∆nkuA::argB*, *pyrG89*, *pyroA4*, *veA^+^*, *velB^L61A^:sgfp:six*	This study
AGB1485	*∆nkuA::argB*, *pyrG89*, *pyroA4*, *veA^+^*, *velB^Q65A^:sgfp:six*	This study
AGB1486	*∆nkuA::argB*, *pyrG89*, *pyroA4*, *veA^+^*, *velB^P67A^:sgfp:six*	This study
AGB1487	*∆nkuA::argB*, *pyrG89*, *pyroA4*, *veA^+^*, *velB^R71A^:sgfp:six*	This study
AGB1488	*∆nkuA::argB*, *pyrG89*, *pyroA4*, *veA^+^*, *velB^C73A^:sgfp:six*	This study
AGB1489	*∆nkuA::argB*, *pyrG89*, *pyroA4*, *veA^+^*, *velB^G74A^:sgfp:six*	This study
AGB1490	*∆nkuA::argB*, *pyrG89*, *pyroA4*, *veA^+^*, *velB^G76A^:sgfp:six*	This study
AGB1491	*∆nkuA::argB*, *pyrG89*, *pyroA4*, *veA^+^*, *velB^K78A^:sgfp:six*	This study
AGB1492	*∆nkuA::argB*, *pyrG89*, *pyroA4*, *veA^+^*, *velB^D79A^:sgfp:six*	This study
AGB1493	*∆nkuA::argB*, *pyrG89*, *pyroA4*, *veA^+^*, *velB^R80A^:sgfp:six*	This study
AGB1494	*∆nkuA::argB*, *pyrG89*, *pyroA4*, *veA^+^*, *velB^R81A^:sgfp:six*	This study
AGB1495	*∆nkuA::argB*, *pyrG89*, *pyroA4*, *veA^+^*, *velB^P82A^:sgfp:six*	This study
AGB1496	*∆nkuA::argB*, *pyrG89*, *pyroA4*, *veA^+^*, *velB^P85A^:sgfp:six*	This study
AGB1497	*∆nkuA::argB*, *pyrG89*, *pyroA4*, *veA^+^*, *velB^P86A^:sgfp:six*	This study
AGB1498	*∆nkuA::argB*, *pyrG89*, *pyroA4*, *veA^+^*, *velB^P87A^:sgfp:six*	This study
AGB1499	*∆nkuA::argB*, *pyrG89*, *pyroA4*, *veA^+^*, *velBcDNA(∆velB_DBR::CapVelvet_DBR)^R71^:sgfp:six*	This study

Note: Most strains were generated via recyclable selection marker cassettes. Upon marker recycling from the host genome, this system leaves only a minimal residual scar sequence of approximately 100 nucleotides at the target locus. For strain AGB1499, the designation “R71” denotes an arginine residue insertion at position 71. Abbreviations: CapVelvet, velvet gene from *Capsaspora owczarzaki*; DBR, DNA-binding region.

*E. coli* strains were grown on solid lysogeny broth (LB) medium (1% tryptone, 0.5% yeast extract, and 1% NaCl) or in liquid LB at 37°C with shaking at 200 rpm (Bertani 1951). Ampicillin (100 µg/mL) or kanamycin (50 µg/mL) was added to prevent plasmid loss.

### Plasmid and strain preparation

Plasmids were amplified in *E. coli* and extracted using the Plasmid Spin Miniprep Kit (QIAGEN, Germany). Plasmid transformation into *E. coli* was carried out following established protocols (Inoue et al. 1990; Hanahan et al. 1991). Positive *E. coli* transformants on the selection medium were identified via colony polymerase chain reaction (PCR) and plasmid sequencing. All plasmids used in the present study are summarized in Suppl. material 1: table SS1. The oligonucleotides used in the construction of plasmids are listed in Suppl. material 1: table SS2.

*A. nidulans* transformation was conducted via polyethylene glycol-mediated protoplast fusion according to published methods (Punt and van den Hondel 1992; Eckert et al. 2000). All transforming plasmids carrying recyclable phleomycin-resistance cassettes are presented in Suppl. material 1: table SS1. These plasmids were linearized by restriction digestion with PmeI, which possesses two recognition sites on each vector. The purified target fragments (5 μg) were incubated with *A. nidulans* protoplasts. Successful genomic integration was validated by Southern blot hybridization using the AlkPhos Direct Labelling and Detection System (GE Healthcare Life Technologies, UK). All mutant strains generated herein are listed in Table 1.

### Construction of plasmid pME5335 and strains with replacement of *velB* DNA-binding region by *C. owczarzaki* velvet one in *A. nidulans* (AGB1481)

The *velB* 5’ untranslated region (UTR) and 5’ fragment, cloned with primers WC30 and WC154 from template pME5333, and the remaining part of *velB* cDNA fused with *sgfp*, cloned from pME5333 with primers WC12 and WC155, were integrated into the SwaI restriction site of plasmid pME5332 to generate pME5335, employing the Seamless Cloning and Assembly Kit (Invitrogen, Thermo Fisher Scientific, USA). The DNA sequence encoding the *C. owczarzaki* velvet DNA-binding region (amino acid sequence: LHIRQQPKHACMGGVSHDGARGNRR) was incorporated into the recombinant construct through primer-directed mutagenesis using primers WC154 and WC155. The linear *velBcDNA*(*∆velB_DBR*::*CapVelvet_DBR*):sgfp: phleoRM cassette excised from pME5335 by PmeI was integrated into AGB1064, resulting in AGB1481.

### Construction of plasmid pME5338 and *A. nidulans* mutant of *velB^L61A^* (AGB1484)

To generate the pME5338 recombinant plasmid, two targeted DNA fragments were amplified from the pME4687 template. The first fragment covering the *velB* 5' UTR and its partial 5' coding region was amplified using the primer pair WC30/WC208, while the second fragment containing the residual 3' terminal sequence of *velB* fused to the *sgfp* reporter gene was amplified with WC12/WC209. These two fragments were seamlessly inserted into the SwaI restriction site of the pME5332 backbone utilizing the Seamless Cloning and Assembly Kit (Invitrogen, Thermo Fisher Scientific, USA). The L61A point mutation was incorporated into the *velB* coding region via primer-mediated mutagenesis using primers WC208 and WC209. The linear expression cassette *velB^L61A^: sgfp: phleoRM* was linearized from pME5338 through PmeI restriction digestion and transformed into the recipient *A. nidulans* strain AGB1064, yielding the final mutant strain AGB1484.

### Construction of plasmid pME5339 and *A. nidulans* mutant of *velB^Q65A^* (AGB1485)

To generate the pME5339 recombinant plasmid, two targeted DNA fragments were amplified from the pME4687 template. The first fragment covering the *velB* 5' UTR and its partial 5' coding region was amplified using the primer pair WC30/WC171, while the second fragment containing the residual 3' terminal sequence of *velB* fused to the *sgfp* gene was amplified with WC12/WC172. These two fragments were seamlessly inserted into the SwaI restriction site of the pME5332 backbone utilizing a Seamless Cloning and Assembly Kit (Invitrogen, Thermo Fisher Scientific, USA). The Q65A point mutation was incorporated into the *velB* coding region via primer-mediated mutagenesis using primers WC171 and WC172. Subsequently, the linear expression cassette *velB^Q65A^: sgfp: phleoRM* was excised from pME5339 through PmeI restriction digestion and transformed into the recipient *A. nidulans* strain AGB1064, yielding the mutant strain AGB1485.

### Construction of plasmid pME5340 and *A. nidulans* mutant of *velB^P67A^* (AGB1486)

To generate the pME5340 recombinant plasmid, two targeted DNA fragments were amplified from the pME4687 template. The first fragment covering the *velB* 5' UTR and its partial 5' coding region was amplified using the primer pair WC30/WC173, while the second fragment containing the residual 3' terminal sequence of *velB* fused to the *sgfp* gene was amplified with WC12/WC174. These two fragments were seamlessly inserted into the SwaI restriction site of the pME5332 backbone utilizing a Seamless Cloning and Assembly Kit (Invitrogen, Thermo Fisher Scientific, USA). The P67A point mutation was incorporated into the *velB* coding region via primer-mediated mutagenesis using primers WC173 and WC174. Subsequently, the linear expression cassette *velB^P67A^: sgfp: phleoRM* was excised from pME5340 through PmeI restriction digestion and transformed into the recipient *A. nidulans* strain AGB1064, yielding the mutant strain AGB1486.

### Construction of plasmid pME5341 and *A. nidulans* mutant of *velB^R71A^* (AGB1487)

To generate the pME5341 recombinant plasmid, two targeted DNA fragments were amplified from the pME4687 template. The first fragment covering the *velB* 5' UTR and its partial 5' coding region was amplified using the primer pair WC30/WC175, while the second fragment containing the residual 3' terminal sequence of *velB* fused to the *sgfp* gene was amplified with WC12/WC176. These two fragments were seamlessly inserted into the SwaI restriction site of the pME5332 backbone utilizing a Seamless Cloning and Assembly Kit (Invitrogen, Thermo Fisher Scientific, USA). The R71A point mutation was incorporated into the *velB* coding region via primer-mediated mutagenesis using primers WC175 and WC176. Subsequently, the linear expression cassette *velB^R71A^: sgfp: phleoRM* was excised from pME5341 through PmeI restriction digestion and transformed into the recipient *A. nidulans* strain AGB1064, yielding the mutant strain AGB1487.

### Construction of plasmid pME5342 and *A. nidulans* mutant of *velB^C73A^* (AGB1488)

To generate the pME5342 recombinant plasmid, two targeted DNA fragments were amplified from the pME4687 template. The first fragment covering the *velB* 5' UTR and its partial 5' coding region was amplified using the primer pair WC30/WC251, while the second fragment containing the residual 3' terminal sequence of *velB* fused to the *sgfp* gene was amplified with WC12/WC252. These two fragments were seamlessly inserted into the SwaI restriction site of the pME5332 backbone utilizing a Seamless Cloning and Assembly Kit (Invitrogen, Thermo Fisher Scientific, USA). The C73A point mutation was incorporated into the *velB* coding region via primer-mediated mutagenesis using primers WC251 and WC252. Subsequently, the linear expression cassette *velB^C73A^: sgfp: phleoRM* was excised from pME5342 through PmeI restriction digestion and transformed into the recipient *A. nidulans* strain AGB1064, yielding the mutant strain AGB1488.

### Construction of plasmid pME5343 and *A. nidulans* mutant of *velB^G74A^* (AGB1489)

To generate the pME5343 recombinant plasmid, two targeted DNA fragments were amplified from the pME4687 template. The first fragment covering the *velB* 5' UTR and its partial 5' coding region was amplified using the primer pair WC30/WC177, while the second fragment containing the residual 3' terminal sequence of *velB* fused to the *sgfp* gene was amplified with WC12/WC178. These two fragments were seamlessly inserted into the SwaI restriction site of the pME5332 backbone utilizing a Seamless Cloning and Assembly Kit (Invitrogen, Thermo Fisher Scientific, USA). The G74A point mutation was incorporated into the *velB* coding region via primer-mediated mutagenesis using primers WC177 and WC178. Subsequently, the linear expression cassette *velB^G74A^: sgfp: phleoRM* was excised from pME5343 through PmeI restriction digestion and transformed into the recipient *A. nidulans* strain AGB1064, yielding the mutant strain AGB1489.

### Construction of plasmid pME5344 and *A. nidulans* mutant of *velB^G76A^* (AGB1490)

To generate the pME5344 recombinant plasmid, two targeted DNA fragments were amplified from the pME4687 template. The first fragment covering the *velB* 5' UTR and its partial 5' coding region was amplified using the primer pair WC30/WC210, while the second fragment containing the residual 3' terminal sequence of *velB* fused to the *sgfp* gene was amplified with WC12/WC211. These two fragments were seamlessly inserted into the SwaI restriction site of the pME5332 backbone utilizing a Seamless Cloning and Assembly Kit (Invitrogen, Thermo Fisher Scientific, USA). The G76A point mutation was incorporated into the *velB* coding region via primer-mediated mutagenesis using primers WC210 and WC211. Subsequently, the linear expression cassette *velB^G76A^: sgfp: phleoRM* was excised from pME5344 through PmeI restriction digestion and transformed into the recipient *A. nidulans* strain AGB1064, yielding the mutant strain AGB1490.

### Construction of plasmid pME5345 and *A. nidulans* mutant of *velB^K78A^* (AGB1491)

To generate the pME5345 recombinant plasmid, two targeted DNA fragments were amplified from the pME4687 template. The first fragment covering the *velB* 5' UTR and its partial 5' coding region was amplified using the primer pair WC30/WC179, while the second fragment containing the residual 3' terminal sequence of *velB* fused to the *sgfp* gene was amplified with WC12/WC180. These two fragments were seamlessly inserted into the SwaI restriction site of the pME5332 backbone utilizing a Seamless Cloning and Assembly Kit (Invitrogen, Thermo Fisher Scientific, USA). The K78A point mutation was incorporated into the *velB* coding region via primer-mediated mutagenesis using primers WC179 and WC180. Subsequently, the linear expression cassette *velB^K78A^: sgfp: phleoRM* was excised from pME5345 through PmeI restriction digestion and transformed into the recipient *A. nidulans* strain AGB1064, yielding the mutant strain AGB1491.

### Construction of plasmid pME5346 and *A. nidulans* mutant of *velB^D79A^* (AGB1492)

To generate the pME5346 recombinant plasmid, two targeted DNA fragments were amplified from the pME4687 template. The first fragment covering the *velB* 5' UTR and its partial 5' coding region was amplified using the primer pair WC30/WC181, while the second fragment containing the residual 3' terminal sequence of *velB* fused to the *sgfp* gene was amplified with WC12/WC182. These two fragments were seamlessly inserted into the SwaI restriction site of the pME5332 backbone utilizing a Seamless Cloning and Assembly Kit (Invitrogen, Thermo Fisher Scientific, USA). The D79A point mutation was incorporated into the *velB* coding region via primer-mediated mutagenesis using primers WC181 and WC182. Subsequently, the linear expression cassette *velB^D79A^: sgfp: phleoRM* was excised from pME5346 through PmeI restriction digestion and transformed into the recipient *A. nidulans* strain AGB1064, yielding the mutant strain AGB1492.

### Construction of plasmid pME5347 and *A. nidulans* mutant of *velB^R80A^* (AGB1493)

To generate the pME5347 recombinant plasmid, two targeted DNA fragments were amplified from the pME4687 template. The first fragment covering the *velB* 5' UTR and its partial 5' coding region was amplified using the primer pair WC30/WC183, while the second fragment containing the residual 3' terminal sequence of *velB* fused to the *sgfp* gene was amplified with WC12/WC184. These two fragments were seamlessly inserted into the SwaI restriction site of the pME5332 backbone utilizing a Seamless Cloning and Assembly Kit (Invitrogen, Thermo Fisher Scientific, USA). The R80A point mutation was incorporated into the *velB* coding region via primer-mediated mutagenesis using primers WC183 and WC184. Subsequently, the linear expression cassette *velB^R80A^: sgfp: phleoRM* was excised from pME5347 through PmeI restriction digestion and transformed into the recipient *A. nidulans* strain AGB1064, yielding the mutant strain AGB1493.

### Construction of plasmid pME5348 and *A. nidulans* mutant of *velB^R81A^* (AGB1494)

To generate the pME5348 recombinant plasmid, two targeted DNA fragments were amplified from the pME4687 template. The first fragment covering the *velB* 5' UTR and its partial 5' coding region was amplified using the primer pair WC30/WC185, while the second fragment containing the residual 3' terminal sequence of *velB* fused to the *sgfp* gene was amplified with WC12/WC186. These two fragments were seamlessly inserted into the SwaI restriction site of the pME5332 backbone utilizing a Seamless Cloning and Assembly Kit (Invitrogen, Thermo Fisher Scientific, USA). The R81A point mutation was incorporated into the *velB* coding region via primer-mediated mutagenesis using primers WC185 and WC186. Subsequently, the linear expression cassette *velB^R81A^: sgfp: phleoRM* was excised from pME5348 through PmeI restriction digestion and transformed into the recipient *A. nidulans* strain AGB1064, yielding the mutant strain AGB1494.

### Construction of plasmid pME5349 and *A. nidulans* mutant of *velB^P82A^* (AGB1495)

To generate the pME5349 recombinant plasmid, two targeted DNA fragments were amplified from the pME4687 template. The first fragment covering the *velB* 5' UTR and its partial 5' coding region was amplified using the primer pair WC30/WC187, while the second fragment containing the residual 3' terminal sequence of *velB* fused to the *sgfp* gene was amplified with WC12/WC188. These two fragments were seamlessly inserted into the SwaI restriction site of the pME5332 backbone utilizing a Seamless Cloning and Assembly Kit (Invitrogen, Thermo Fisher Scientific, USA). The P82A point mutation was incorporated into the *velB* coding region via primer-mediated mutagenesis using primers WC187 and WC188. Subsequently, the linear expression cassette *velB^P82A^: sgfp: phleoRM* was excised from pME5349 through PmeI restriction digestion and transformed into the recipient *A. nidulans* strain AGB1064, yielding the mutant strain AGB1495.

### Construction of plasmid pME5350 and *A. nidulans* mutant of *velB^P85A^* (AGB1496)

To generate the pME5350 recombinant plasmid, two targeted DNA fragments were amplified from the pME4687 template. The first fragment covering the *velB* 5' UTR and its partial 5' coding region was amplified using the primer pair WC30/WC189, while the second fragment containing the residual 3' terminal sequence of *velB* fused to the *sgfp* gene was amplified with WC12/WC190. These two fragments were seamlessly inserted into the SwaI restriction site of the pME5332 backbone utilizing a Seamless Cloning and Assembly Kit (Invitrogen, Thermo Fisher Scientific, USA). The P85A point mutation was incorporated into the *velB* coding region via primer-mediated mutagenesis using primers WC189 and WC190. Subsequently, the linear expression cassette *velB^P85A^: sgfp: phleoRM* was excised from pME5350 through PmeI restriction digestion and transformed into the recipient *A. nidulans* strain AGB1064, yielding the mutant strain AGB1496.

### Construction of plasmid pME5351 and *A. nidulans* mutant of *velB^P86A^* (AGB1497)

To generate the pME5351 recombinant plasmid, two targeted DNA fragments were amplified from the pME4687 template. The first fragment covering the *velB* 5' UTR and its partial 5' coding region was amplified using the primer pair WC30/WC191, while the second fragment containing the residual 3' terminal sequence of *velB* fused to the *sgfp* gene was amplified with WC12/WC192. These two fragments were seamlessly inserted into the SwaI restriction site of the pME5332 backbone utilizing a Seamless Cloning and Assembly Kit (Invitrogen, Thermo Fisher Scientific, USA). The P86A point mutation was incorporated into the *velB* coding region via primer-mediated mutagenesis using primers WC191 and WC192. Subsequently, the linear expression cassette *velB^P86A^: sgfp: phleoRM* was excised from pME5351 through PmeI restriction digestion and transformed into the recipient *A. nidulans* strain AGB1064, yielding the mutant strain AGB1497.

### Construction of plasmid pME5352 and *A. nidulans* mutant of *velB^P87A^* (AGB1498)

To generate the pME5352 recombinant plasmid, two targeted DNA fragments were amplified from the pME4687 template. The first fragment covering the *velB* 5' UTR and its partial 5' coding region was amplified using the primer pair WC30/WC193, while the second fragment containing the residual 3' terminal sequence of *velB* fused to the *sgfp* gene was amplified with WC12/WC194. These two fragments were seamlessly inserted into the SwaI restriction site of the pME5332 backbone utilizing a Seamless Cloning and Assembly Kit (Invitrogen, Thermo Fisher Scientific, USA). The P87A point mutation was incorporated into the *velB* coding region via primer-mediated mutagenesis using primers WC193 and WC194. Subsequently, the linear expression cassette *velB^P87A^: sgfp: phleoRM* was excised from pME5352 through PmeI restriction digestion and transformed into the recipient *A. nidulans* strain AGB1064, yielding the mutant strain AGB1498.

### Construction of plasmid pME5353 and *A. nidulans* mutant of *velBcDNA*(*∆velB_DBR::CapVelvet_DBR)^R71^* (AGB1499)

The *velB* 5’ UTR and 5’ fragment cloned with primers WC30 and WC212, and the remaining 3’ fragment fused with *sgfp* cloned with primers WC12 and WC213 from template pME5335, were integrated into the SwaI restriction cutting site of plasmid pME5332 to generate pME5353, employing the Seamless Cloning and Assembly Kit (Invitrogen, Thermo Fisher Scientific, USA). The mutant *velBcDNA(∆velB_DBR::CapVelvet_DBR)^R71^* was introduced by primers WC212 and WC213. The linear *velBcDNA(∆velB_DBR::CapVelvet_DBR)^R71^*: *sgfp*: *phleoRM* cassette obtained by digestion of pME5353 with PmeI was integrated into AGB1064, resulting in AGB1499.

### Sequence logo creation of DNA-binding domains

Multiple protein sequence alignments corresponding to the DNA-binding domains of six transcription factor families, including Rel (PF00554), zinc finger (PF01530), homeobox (PF00046), basic region leucine zipper (PF00170), SRF-TF (PF00319), and helix-loop-helix (PF00010), were retrieved from the InterPro database (Blum et al. 2025; Paysan-Lafosse et al. 2025). Multiple sequence alignment of velvet DNA-binding domains was performed in accordance with a previously reported protocol (Chen et al. 2025). Briefly, a total of 4,999 putative velvet proteins across the fungal kingdom were obtained from the published dataset (Chen et al. 2025), and then aligned by HMMER (version 3.0, http://hmmer.org/) (Mistry et al. 2013) against the seed hidden Markov model of the velvet domain (PF11754) downloaded from the InterPro database (https://www.ebi.ac.uk/interpro/entry/pfam/PF11754/) (Blum et al. 2025; Paysan-Lafosse et al. 2025). Conserved sequence logos based on the aforementioned alignments were generated using WebLogo 3 (Crooks et al. 2004). Subsequent sequence editing and statistical analyses were implemented via Jalview Desktop (version 2.11.5.1) (Waterhouse et al. 2009).

### Protein isolation of *A. nidulans* mycelia

Protein extraction from *A. nidulans* mycelia was conducted following a previously described protocol (Thieme et al. 2018). Briefly, *A. nidulans* strains were cultivated vegetatively in 500 mL MM inoculated with 5 × 10^8^ spores at 37°C for 20 h. The harvested mycelia were collected using sterile filters (Merck, Darmstadt, Germany), rinsed with saline-PMSF-DMSO, dehydrated, and immediately snap-frozen in liquid nitrogen. Frozen mycelial samples were homogenized with a table mill under liquid nitrogen conditions. The pulverized tissues were resuspended in an equal volume of B+ buffer (300 mM NaCl, 100 mM Tris, pH 7.5, 10% glycerol, 1 mM EDTA, 0.1% NP-40). The buffer was presupplemented with 1.5 mM DTT, complete EDTA-free protease inhibitor cocktail (ROCHE Diagnostics GmbH, Basel, Switzerland), and 0.001 mM PMSF. The resulting homogenate was centrifuged at 13,000 rpm for 30 min at 4°C. The clarified supernatant was aliquoted into new centrifuge tubes and stored at −20°C. The protein concentration of each sample was quantified using a NanoDrop ND-1000 spectrophotometer.

### Western blot analysis of *A. nidulans* protein extracts

GFP fusion proteins were detected via western blotting based on a previously established method (Chen et al. 2025). Briefly, crude protein samples were denatured with 3× protein loading buffer composed of 250 mM Tris-HCl (pH 6.8), 15% (v/v) β-mercaptoethanol, 30% (v/v) glycerol, 7% (v/v) SDS, and 0.3% (w/v) bromophenol blue. Protein samples were subjected to thermal denaturation at 95°C for 5 min. Equal amounts of denatured crude protein extracts (90 µg per lane) from each fungal strain were resolved by sodium dodecyl sulfate-polyacrylamide gel electrophoresis (SDS-PAGE) using 12% polyacrylamide gels. Separated proteins were electrophoretically transferred onto nitrocellulose membranes (Merck, Darmstadt, Germany) at a constant voltage of 100 V for 1 h.

Membranes were blocked with 5% (w/v) skim milk powder dissolved in Tris-buffered saline containing 0.05% Tween 20 (TBST; 10 mM Tris-HCl, pH 8.0, 150 mM NaCl) for 1 h at room temperature. The blocked membranes were then incubated with a mouse monoclonal anti-GFP primary antibody (sc-9996, Santa Cruz Biotechnology) diluted 1:250 in blocking buffer. Following three consecutive washes with TBST, membranes were incubated with a horseradish peroxidase-conjugated goat anti-mouse secondary antibody (115-035-003, Jackson ImmunoResearch, West Grove, USA) at a 1:1,000 dilution for 1 h with gentle agitation at room temperature. Membranes were subsequently washed three additional times with TBST to remove unbound secondary antibody.

Chemiluminescent detection was performed using an enhanced chemiluminescence (ECL) reagent prepared immediately before use. Solution A consisted of 9 mL deionized water, 1 mL 1 M Tris-HCl (pH 8.5), 100 µL 250 mM luminol, and 44 µL 400 mM *p*-coumaric acid. Solution B contained 9 mL deionized water, 1 mL 1 M Tris-HCl (pH 8.5), and 6.14 µL 30% hydrogen peroxide. Equal volumes of Solution A and Solution B were mixed and applied to the membrane, which was then incubated for 2 min in the dark with gentle rocking. Chemiluminescent signals were captured using the Fusion-SL7 imaging system (Vilber Lourmat) and quantified using Fusion software (version 15.18) and Bio1D software (version 15.08).

### Extraction of secondary metabolites and their LC-MS analysis

Extraction of secondary metabolites and subsequent LC-MS analysis were performed as described previously (Chen et al. 2025). Briefly, each fungal strain was inoculated with 1 × 10^6^ fresh spores on solid MM supplemented with 0.1% pyridoxine-HCl, 5 mM uridine, and 5 mM uracil and incubated in the dark at 37°C for 7 days. Metabolites were extracted from two colony agar plugs by homogenization in 5 mL water and 5 mL ethyl acetate, followed by overnight shaking at 220 rpm. After centrifugation at 2,500 rpm for 5 min, the ethyl acetate phase was collected, evaporated to dryness, and stored at −20°C. All extractions were performed in triplicate.

For LC-MS analysis, extracts were reconstituted in 500 µL acetonitrile/water (1:1) and centrifuged at 13,000 rpm for 10 min at 4°C. Chromatographic separation was performed on an Acclaim 120 C18 column (4.6 × 100 mm, 5 µm) using a 0.1% formic acid/acetonitrile gradient (5–95% B in 20 min, 10 min isocratic wash) at 0.8 mL/min and 30°C. Mass spectra were acquired in positive and negative ESI modes (*m/z* 70–1050) on a Q Exactive Focus Orbitrap mass spectrometer coupled to an UltiMate 3000 HPLC system. Data were analyzed using Xcalibur 4.4 and FreeStyle 1.8 SP2 software.

### Conidiospore and cleistothecia quantification

Fresh conidia were harvested from 3-day-old fungal cultures grown on solid MM plates using sterile 0.96% (w/v) NaCl solution supplemented with 0.002% (v/v) Tween 80. The resulting conidial suspensions were filtered through sterile Miracloth to remove residual mycelial debris, and the filtrates were used as conidial stock solutions. Stock suspensions were diluted to the required concentrations with sterile distilled water as needed. Conidial concentrations were quantified using a Coulter Z2 particle counter (Beckman Coulter GmbH, Krefeld, Germany).

Cleistothecia quantification was performed as described previously (Thieme et al. 2018). Briefly, 5 mm-diameter agar plugs (excised using the wide end of a standard 200 μL pipette tip) were harvested from representative regions of mature colonies. Cleistothecia were gently teased apart and dispersed onto a fresh sterile agar plate to ensure complete separation of individual fruiting bodies. Cleistothecial counts were determined using an SZX12-ILLB2-200 stereomicroscope (Olympus Corporation, Tokyo, Japan). All experiments were performed in biological triplicate.

### Spore viability assay

Spore viability assays were performed as described previously (Chen et al. 2025). Briefly, fresh conidia were harvested from 3-day-old fungal cultures grown on solid minimal medium (MM) agar plates and resuspended in sterile 0.96% (w/v) NaCl solution supplemented with 0.002% (v/v) Tween 80. Conidial suspensions were filtered through sterile Miracloth to remove mycelial debris and serially diluted to a final concentration of 1 × 10^6^ conidia/mL. Stock suspensions were stored at 4°C in the dark until use.

For viability assessment at designated time points post-harvest, stock spore solutions were further serially diluted. Aliquots (100 μL) containing approximately 100 conidia were spread evenly onto solid MM agar plates and incubated at 37°C under constant light for 2 days. Colony-forming units (CFUs) were counted manually. All assays were performed in three independent biological replicates.

Spore viability was calculated as the percentage of viable conidia relative to the initial fresh spore inoculum, using the formula:

$$\text{Viability}(\%)=\left(\frac{\text{number of CFUs after treatment}}{\text{number of CFUs from untreated fresh spores}}\right)\times 100$$

Statistical significance was determined using unpaired two-tailed Student’s *t*-tests, comparing the mean values of indicated mutant strains to the wild-type control. Data are presented as mean ± standard deviation (SD) of three independent experiments.

### Microscopy

Representative colonies of *A. nidulans* were imaged using an SZX12-ILLB2-200 stereomicroscope (Olympus Corporation, Tokyo, Japan). Digital images were captured with an Olympus SC30 digital camera coupled to the stereomicroscope, and post-acquisition image processing was performed using cellSens software (Olympus).

### Structural modeling and analysis of VelB and related variants

Three-dimensional structures of VelB and its derived variants VelB^R71A^, VelB^R80A^, and VelB^R81A^ were predicted using AlphaFold 3 (Abramson et al. 2024). The crystal structure of the *A. nidulans* VosA–VelB heterodimer (PDB ID: 4N6R) (Ahmed et al. 2013) was employed as the reference structural template for modeling. The highest-confidence model generated by AlphaFold 3 was used for further analysis.

Per-residue electrostatic potential values of the VelB, VelB^R71A^, VelB^R80A^, and VelB^R81A^ structures were calculated using the Coulomb Potential model (Honig and Nicholls 1995). Briefly, the protein structures in mmCIF format were parsed to extract the Cartesian coordinates (*x*, *y*, *z*) and identity of all heavy atoms. Partial atomic charges were assigned using a residue-level net charge model based on the AMBER ff14SB force field (Maier et al. 2015). The electrostatic potential at each atomic position was computed using the Coulomb potential with a uniform dielectric medium:

$$\phi_{i}=\frac{332.0637}{\varepsilon_{r}}\sum_{j\neq i}\frac{q_{j}}{r_{ij}}$$

where *Φ_i_* is the electrostatic potential at atom *i* (in kcal/(mol·e)), *q_j_* is the partial charge of atom *j* (in units of elementary charge, *e*), *r_ij_* is the interatomic distance between atoms *i* and *j* (in Angstroms, A), *ε_r_* = 78.5 is the dielectric constant of water at 310 K, and the constant 332.0637 converts the expression to units of kcal/(mol·e). The summation extends over all atoms *j* except the atom *i* itself.

The Coulomb potentials were averaged over all heavy atoms belonging to each amino acid residue to obtain a per-residue electrostatic potential value. All calculations were performed using custom Python scripts (version 3.10) with NumPy (version 1.24) for numerical computation. Calculations were executed on a standard computing workstation. The complete source code is available upon request.

## Results

### Sequence features of velvet DNA-binding region and alanine scanning of conserved residues in *A. nidulans* VelB

**Figure 1.**
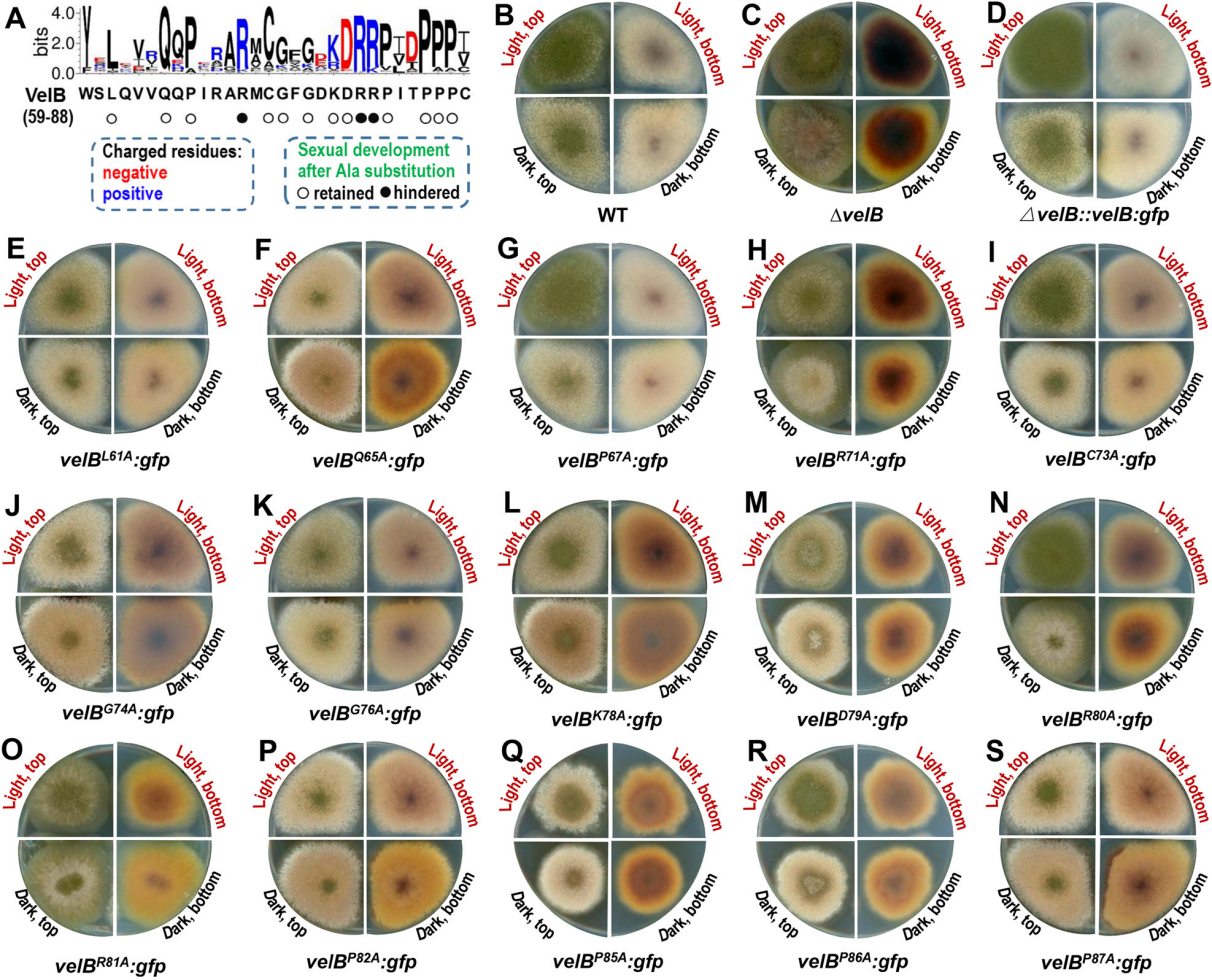
Alanine scanning of 15 conserved residues in the velvet DNA-binding region of *A. nidulans* VelB. **A**. Sequence logo of 4,999 velvet DNA-binding regions and mutagenesis summary. Residues are colored by charge (blue: positive; red: negative; black: neutral). **B–S**. Colony morphology of strains cultured on MM plates at 37°C for 5 days under asexual (light) and sexual (dark) conditions. **B–D**. Respectively correspond to the wild type (WT, AGB551), *velB* knockout (AGB1064), and *velB* complement (AGB1192), which were used for comparison with the mutants. **E–S**. Respectively correspond to *velB^L61A^:gfp* (AGB1484), *velB^Q65A^:gfp* (AGB1485), *velB^P67A^:gfp* (AGB1486), *velB^R71A^:gfp* (AGB1487), *velB^C73A^:gfp* (AGB1488), *velB^G74A^:gfp* (AGB1489), *velB^G76A^:gfp* (AGB1490), *velB^K78A^:gfp* (AGB1491), *velB^D79A^:gfp* (AGB1492), *velB^R80A^:gfp* (AGB1493), *velB^R81A^:gfp* (AGB1494), *velB^P82A^:gfp* (AGB1495), *velB^P85A^:gfp* (AGB1496), *velB^P86A^:gfp* (AGB1497), and *velB^P87A^:gfp* (AGB1498).

*In silico* analysis of amino acid conservation within the velvet DNA-binding domain was performed using 4,999 putative velvet domains derived from diverse fungal species, and the corresponding sequence logo was constructed (Fig. 1A). The results revealed that the velvet DNA-binding domain consists of approximately 30 amino acid residues, among which multiple residues exhibit strong evolutionary conservation. Notably, several positively charged amino acids were presumed to be functionally critical.

To identify the key residues governing the biological functions of velvet proteins, alanine-scanning mutagenesis of 15 conserved residues (conservation score > 1.5 bits) in *A. nidulans* VelB was conducted, wherein each target residue was individually substituted with alanine. *Aspergillus nidulans* VelB was selected as the ideal model for investigating the structure–function relationship of velvet family proteins for two reasons: first, of the four velvet homologs in *A. nidulans*, VelB features the shortest amino acid sequence and the highest cross-species conservation across fungi (Chen et al. 2024); second, functional perturbation of VelB induces distinct phenotypic alterations (Chen et al. 2025). The colonial phenotypes of all mutant strains were compared with those of the wild-type (WT), *velB* knockout, and complemented strains (Fig. 1B–S). Preliminary phenotypic characterization indicated that three mutants, namely *velB^R71A^*, *velB^R80A^*, and *velB^R81A^*, displayed the most dramatic phenotypic defects.

### Arginine 71, 80, and 81 of the VelB DNA-binding region are essential for spore viability, sexual development, and secondary metabolism

**Figure 2.**
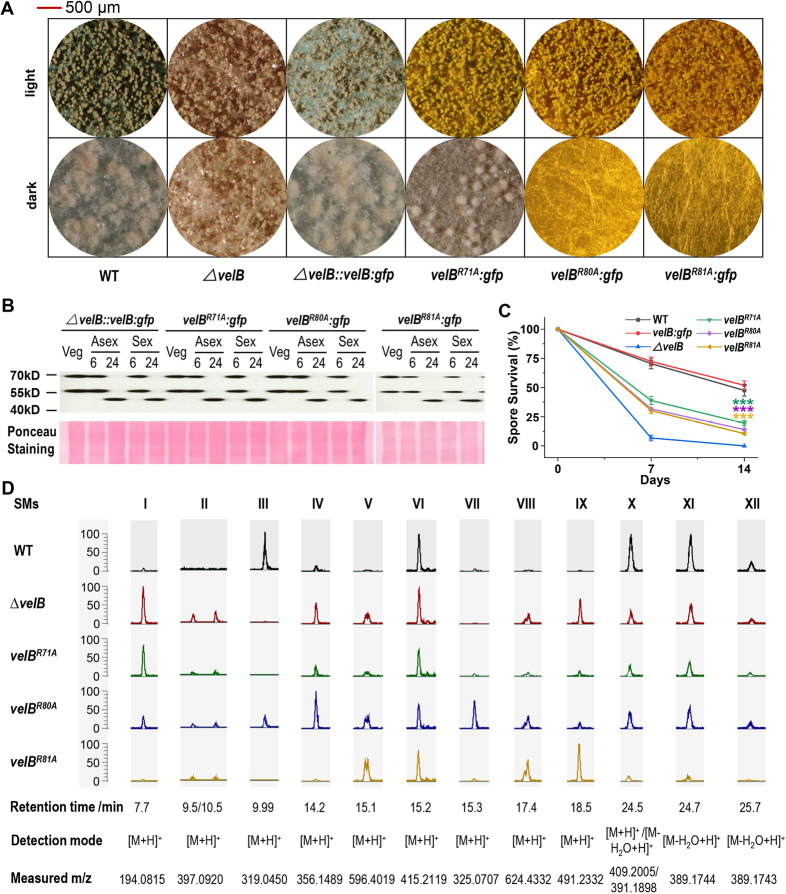
Point mutations of R71A, R80A, or R81A disrupt VelB function and cause defective fungal development in *A. nidulans*. **A**. Fungal developmental phenotypes under asexual (light) and sexual (dark) conditions. The wild type (WT), the *velB* deletion strain, and the VelB-green fluorescent protein (GFP) complementation strain served as controls. Colony morphology was documented on day 5 post-inoculation. **B**. Western blot analysis of VelB^R71A^-GFP, VelB^R80A^-GFP and VelB^R81A^-GFP expression patterns under sexual, asexual, and vegetative growth conditions. The VelB-GFP strain was used as a control. The predicted molecular mass of all full-length fusion proteins is approximately 67 kDa. For all experimental conditions, spores were incubated overnight in liquid MM. For sexual and asexual inductions, mycelia were transferred to solid MM for 6 or 24 h under sexual and asexual conditions, respectively. Ponceau staining was used as a loading control for protein normalization. The VelB-GFP western blot panel was adapted from fig. 2C of the previous study (Chen et al. 2025) under the Creative Commons Attribution 4.0 International license. **C**. Conidial viability assays in the WT, *velB* mutant strains, and complement. A total of 100 conidia per strain were plated after 0, 7, and 14 days of treatment, with initial colony-forming units set to 100%. Error bars represent the standard error of the mean from three independent biological replicates (*** indicates *p* < 0.001 compared to the WT). **D**. SM profiles of WT and *velB* mutant strains. Extracted ion chromatograms of 12 detected SMs are shown. The numbers indicate the identified SMs: I, cichorine (Sanchez et al. 2012); II, asperthecin (Szewczyk et al. 2008); III, F9775A/B (Sanchez et al. 2010); IV, arguosin H (Nielsen et al. 2011); V, emericellamide C (Chiang et al. 2008); VI, austinoneol A (Lo et al. 2012); VII, sterigmatocystin (Yu and Leonard 1995); VIII, emericellamide E (Chiang et al. 2008); IX, terrequinone A (Balibar et al. 2007; Bouhired et al. 2007); X, emericellin (Sanchez et al. 2011); XI, shamixanthone (Sanchez et al. 2011); and XII, epishamixanthone (Sanchez et al. 2011). The chromatogram panels of WT and *velB* knockout strains were adapted from fig. 3B of the previous study (Chen et al. 2025) under the Creative Commons Attribution 4.0 International license.

The impacts of the *velB^R71A^*, *velB^R80A^*, and *velB^R81A^* mutations on fungal development and secondary metabolites were further investigated (Fig. 2). Under asexual growth conditions (in light), *A. nidulans*WT and the *velB*-complemented strain produced green conidia, whereas the *velB*-mutated strains yielded gray-green conidia (Fig. 2A). Under sexual growth conditions (in the dark and sealed), *A. nidulans*WT and the *velB*-complemented strain favored cleistothecia formation; however, the mutated strains *velB^R80A^* and *velB^R81A^* failed to generate cleistothecia on MM, a phenotype identical to that of the *velB* knockout strain (Fig. 2A). The *velB^R71A^* mutant exhibited severely impaired cleistothecia production, with the yield accounting for only 13.3% of that in the WT strain.

The protein expression profiles were compared by western blotting experiments, and the results revealed that, compared to the *velB*:*gfp*-expressing strain, the mutated VelBs were properly expressed (Fig. 2B). The full-length VelB-GFP protein with a molecular weight of 67 kDa was consistently detected across vegetative growth stages, as well as the early developmental stages of both asexual and sexual reproduction. Furthermore, long-term conidial viability was assessed among the WT, *velB* mutants, and complemented strain on solid MM (Fig. 2C). Conidia of the mutated strains *velB^R71A^*, *velB^R80A^*, and *velB^R81A^* exhibited a rapid loss in viability relative to WT conidia after seven days and thereafter. In particular, on the 14^th^ day, the WT and complemented strains maintained a conidial survival rate of approximately 50%. In contrast, the three point-mutant strains displayed a statistically significant reduction in spore viability, with survival rates dropping to roughly 15%. However, unlike the *velB* knockout strain, which completely lost conidial viability after 14 days of treatment, the *velB^R71A^*, *velB^R80A^*, and *velB^R81A^* mutants retained partial conidial viability under identical conditions.

The accumulation patterns of 12 secondary metabolites (SMs) were compared between the *A. nidulans*WT and *velB* mutant strains (Fig. 2D). The *velB* variants exhibited disrupted secondary metabolism, and their metabolic profiles partially overlapped with those of the *velB* deletion strain. Compared with the WT, both the *velB* variants and deletion strain showed attenuated or abolished biosynthesis of emericellin, epishamixanthone, F9775A/B, and shamixanthone. By contrast, the production of asperthecin, emericellamide C, emericellamide E, and terrequinone A was significantly upregulated in these mutant strains. Additionally, austinoneol A displayed stable accumulation levels across all tested WT and *velB* mutant strains. Collectively, these multiple lines of evidence demonstrate that R71, R80, and R81 serve as pivotal functional residues of VelB and are essential for modulating fungal development and secondary metabolism in *A. nidulans*.

### Point mutations of R71A, R80A, and R81A trigger local reduction and global remodeling of electrostatic potential

**Figure 3.**
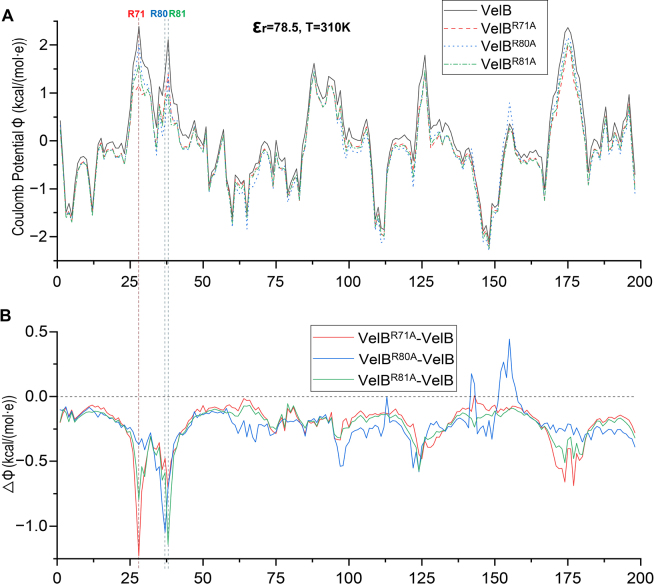
Electrostatic potential dynamics pre- and post-mutation. **A**. Coulomb potentials of VelB, VelB^R71A^, VelB^R80A^, and VelB^R81A^ at each residue. The truncated VelB with 198 residues from the *A. nidulans* VosA–VelB heterodimer (PDB ID: 4N6R) (Ahmed et al. 2013) was employed as the reference structural template for modeling. R71, R80, and R81 correspond to R28, R37, and R38 of the truncated VelB, as indicated in the figure. The modeled structure files of VelB and the R71A, R80A, and R81A variants in CIF format were provided in the supplemental data (Suppl. material 2). **B**. Electrostatic potential changes of VelB^R71A^, VelB^R80A^, and VelB^R81A^ relative to VelB. The value shown at each residue site in the figure represents the difference in electrostatic potential between the mutant VelB and the original VelB. A larger absolute value indicates a greater magnitude of change.

Three positively charged arginine residues (R71, R80, and R81) of the VelB DNA-binding region contribute substantial positive electrostatic potential, which is essential for mediating electrostatic attraction between the protein and the negatively charged DNA molecule. To systematically characterize how mutations at these sites alter the surface electrostatic potential, electrostatic calculations were performed based on the resolved crystal structures of wild-type VelB and its mutant variants. A comparative analysis was conducted (Fig. 3), and core statistical data regarding electrostatic features are summarized in Table 2.

**Table 2.** Key electrostatic statistics among VelB and its variants.

**Parameter**	**VelB**	**VelB^R71A^**	**VelB^R80A^**	**VelB^R81A^**
Coulomb mean (kcal/(mol·e))	+0.0477	–0.1673	–0.1818	–0.1795
Coulomb minimum	–2.106	–2.216	–2.290	–2.246
Coulomb maximum	+2.391	+1.965	+2.158	+2.037
Mutation site/ original potentials		+1.162/+2.391	+0.391/+1.437	+0.976/+2.116
Residues with |ΔΦ| > 0.01		197/198 (99.5%)	197/198 (99.5%)	198/198 (100%)
Residues with |ΔΦ| > 0.05		194/198 (98.0%)	196/198 (98.9%)	198/198 (100%)
Residues with |ΔΦ| > 0.10		155/198 (78.3%)	189/198 (95.5%)	188/198 (94.9%)
Maximum |ΔΦ|		1.229 (Mutation Site)	1.046 (Mutation Site)	1.140 (Mutation Site)

The analysis revealed that, compared to the original VelB, all three mutations significantly decreased the mean, minimum, and maximum Coulomb potential values of the entire protein. R71 is positioned at the strongest electropositive region of VelB, with an original potential of +2.391 kcal/(mol·e). Alanine substitution at this site (R71A) resulted in the most dramatic reduction in electrostatic potential, with an absolute decrease of 1.229 kcal/(mol·e), demonstrating that R71 is a critical residue for maintaining the strong positive electrostatic environment. In contrast, R80 showed the weakest intrinsic positive potential (+1.437 kcal/(mol·e)) among the three arginines. This may have been caused by strong local electrostatic shielding from the nearby negatively charged aspartate residues D77 and D79. Beyond local changes, each mutation triggered extensive global remodeling of the protein electrostatic profile. Further residue-level analysis confirmed that the resultant electrostatic disturbances propagated widely, affecting nearly every amino acid site across the protein structure.

### Replacement of the VelB DNA-binding region by a *Capsaspora* velvet one

**Figure 4.**
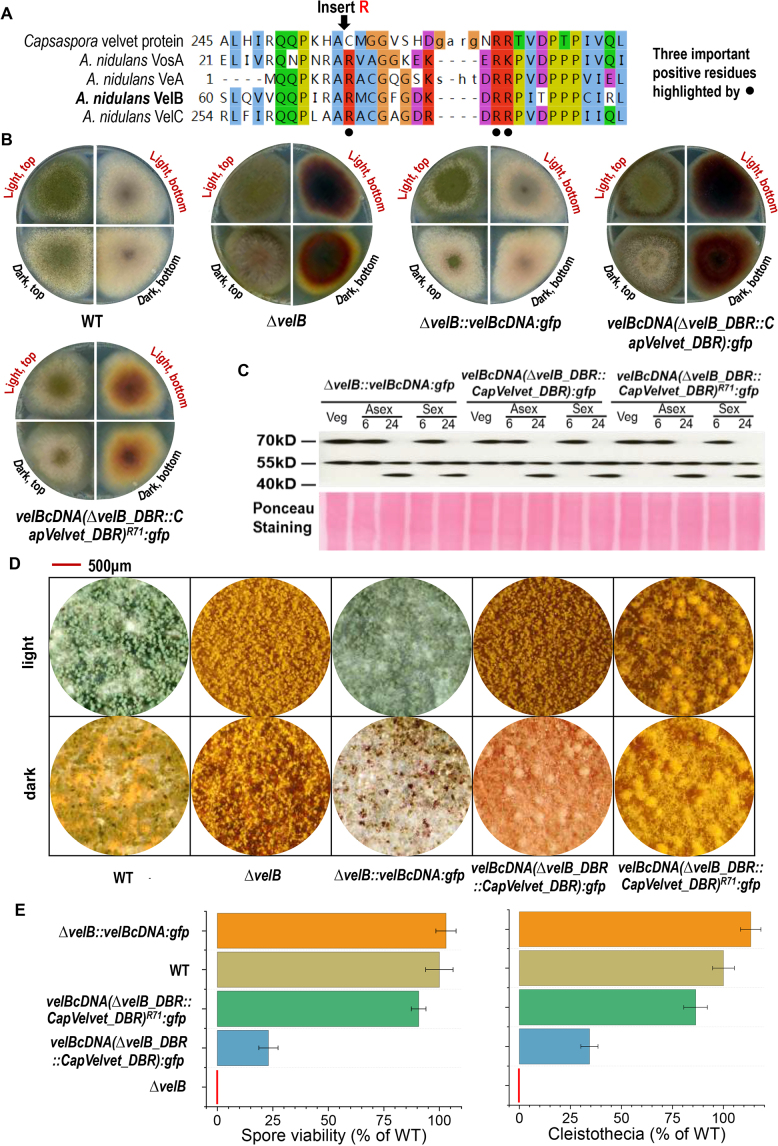
Replacement of the *A. nidulans* VelB DNA-binding region (DBR) by the *Capsaspora* velvet (CapVelvet) region. **A**. Multiple sequence alignment of DNA-binding regions across the *Capsaspora* velvet protein and *A. nidulans* VosA, VeA, VelB, and VelC. Solid black circles denote the three crucial positively charged residues revealed in Fig. 2A. **B**. Colony phenotypic comparison of *A. nidulans*WT, *velB* deletion mutant (Δ*velB*), VelB-GFP complemented strain (expressing C-terminally GFP-tagged *velB* cDNA in the *ΔvelB* background), *velBcDNA(∆velB_DBR::CapVelvet_DBR):gfp*, and *velBcDNA(∆velB_DBR::CapVelvet_DBR)^R71^:gfp*. All strains were grown on solid MM under asexual (light) or sexual (dark, sealed) inducing conditions for 5 days post-inoculation. All mutants were generated using the *A. nidulans velB* cDNA backbone. Notably, the native *Capsaspora* velvet DBR lacks one of the three conserved positively charged residues present in VelB; an arginine residue (R71) was therefore inserted to restore this position, as indicated in Fig. 4A. **C**. Western blot analysis of VelB-GFP and its chimeric/mutant derivatives, all expressed from the native *velB* promoter. Protein detection was performed using an anti-GFP antibody, with Ponceau staining serving as a loading control for protein normalization. Protein abundance was assessed at multiple developmental stages: vegetative growth (24 h submerged culture), asexual development (6 and 24 h light-exposed plates), and sexual development (6 and 24 h dark-incubated plates), all conducted at 37°C. The predicted molecular mass of all full-length fusion proteins is approximately 67 kDa. **D**. Representative micrographs of colonies on day 5 post-inoculation. A red scale bar is provided for size reference. **E**. Conidial viability assays and cleistothecial quantification. Conidial viability of the indicated strains was tested after 14 days of treatment, with viability of the WT strain set to 100%. Cleistothecia of the indicated strains were quantified from plated cultures grown under sexual inducing conditions for 5 days post-inoculation, with cleistothecial production by the WT strain set to 100%. Error bars represent the standard error of the mean from three independent biological replicates.

The velvet protein from *Capsaspora owczarzaki* represents the first identified velvet-family member outside the fungal kingdom (Ahmed et al. 2013). To explore the functional compatibility of the *Capsaspora* velvet DNA-binding region in *A. nidulans* VelB, heterologous domain replacement assays were conducted (Fig. 4). Sequence alignment between the *Capsaspora* velvet DNA-binding region and four *A. nidulans* regions revealed that the former lacks the positively charged arginine residue corresponding to VelB Arg71 (Fig. 4A). Phenotypic assays demonstrated that the chimeric strain *velB*(*∆velB_DBR::CapVelvet_DBR*), in which the native VelB DNA-binding region was substituted with its *Capsaspora* counterpart, failed to fully rescue the phenotypic defects of the *velB* deletion strain. This chimeric mutant only exhibited partial recovery of sexual development under dark culture conditions (Fig. 4B), showing a phenotypic profile highly analogous to that of the mutant *velB^R71A^* described above (Fig. 2A). To verify the indispensable role of R71, this key residue was artificially introduced into the *Capsaspora* velvet DNA-binding region to generate the modified chimeric strain *velB (∆velB_DBR::CapVelvet_DBR)^R71^*. Subsequent phenotypic analyses revealed that supplementation with R71 substantially restored the defective phenotypes of the *velB* knockout strain (Fig. 4B). Notably, all mutants involved in this assay were constructed based on *velB* cDNA to eliminate potential splicing interference. Western blotting was subsequently performed to verify protein expression, and the results confirmed that all chimeric VelB variants were successfully expressed at normal levels relative to the control *velBcDNA:gfp*-expressing strain (Fig. 4C).

The phenotypic characteristics of the two chimeric strains, *velBcDNA(∆velB_DBR::CapVelvet_DBR)*, and *velBcDNA(∆velB_DBR::CapVelvet_DBR)^R71^*, in fungal development and secondary metabolism were further investigated. Under dark, sealed culture conditions for sexual induction, both chimeric mutants were capable of forming cleistothecia on MM (Fig. 4D). The chimeric strain *velBcDNA(∆velB_DBR::CapVelvet_DBR)^R71^* also significantly reduced colony pigmentation levels. Long-term spore viability after 14 days of treatment and cleistothecia amounts were compared among the WT, *velB* mutants, and complemented strain on solid MM (Fig. 4E). For standardized quantitative comparison, the spore viability rate and cleistothecia amount of the WT strain were normalized to 100%, whereas those of the *velB* knockout were set to 0% because of its absence of long-term spore viability and cleistothecia formation. Quantitative data showed that the *velBcDNA(∆velB_DBR::CapVelvet_DBR)* mutant exhibited partial phenotypic restoration, with a 14-day conidial survival rate of approximately 23% and a cleistothecial abundance of 34% relative to the WT. Importantly, the additional introduction of the positively charged residue R71 dramatically enhanced the biological functionality of the chimeric protein. The modified *velB*(*∆velB_DBR::CapVelvet_DBR*) mutant restored conidial viability to 91% and cleistothecial formation to 86% of the WT level, highlighting the essential function of R71 in empowering the conserved DNA-binding region for VelB-mediated physiological regulation.

### Positive residues are frequently present in various DNA-binding regions

**Figure 5.**
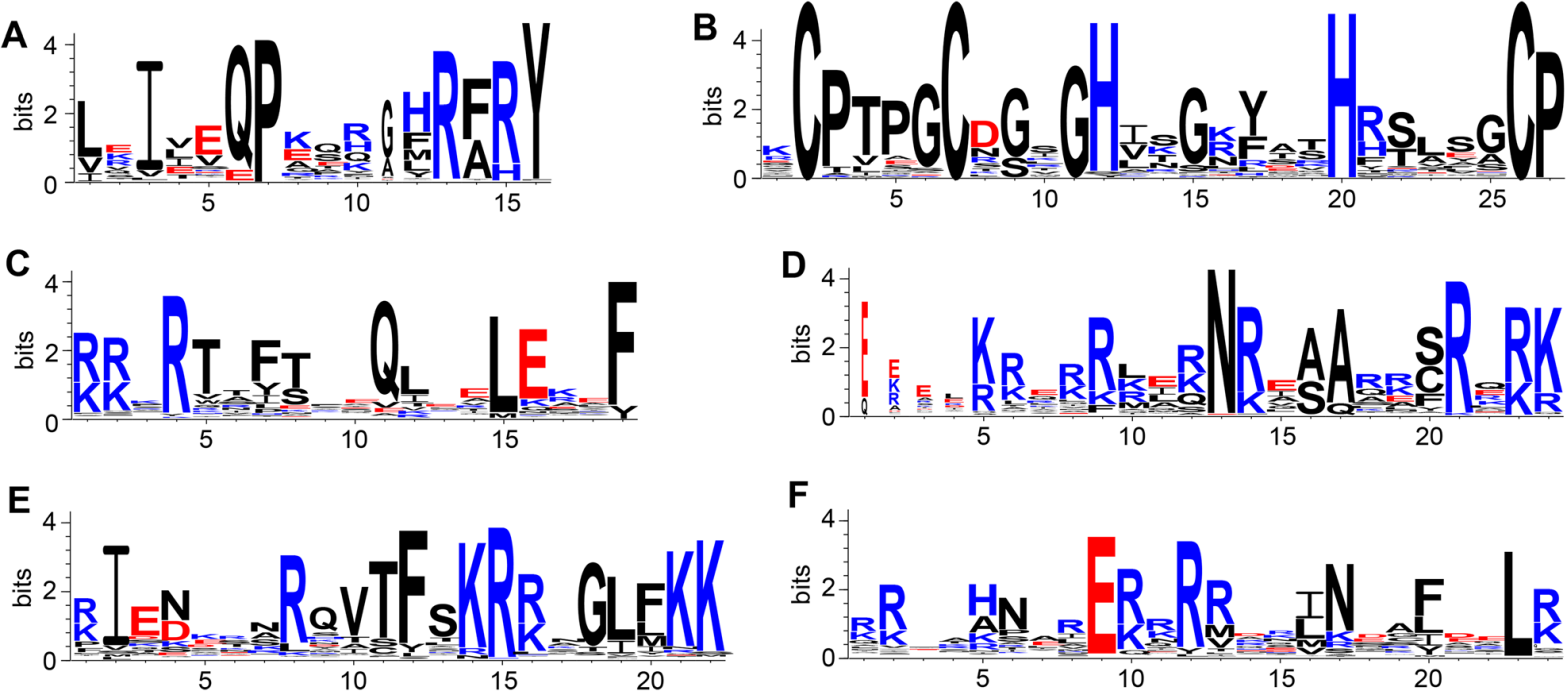
Sequence logo of representative DNA-binding regions. Residues are colored by charge: positive residues are in blue, negative residues are in red, and neutral residues are in black. **A–F**. Correspond to DNA-binding domains of Rel (PF00554), zinc finger (PF01530), homeobox (PF00046), basic region leucine zipper (PF00170), serum response factor (PF00319), and helix-loop-helix (PF00010) transcription factors, respectively.

There are various transcription factor proteins harboring different types of DNA-binding regions. To examine whether positively charged residues are broadly conserved and prevalent across different DNA-binding regions, their sequence features were investigated using the InterPro database (https://www.ebi.ac.uk/interpro). The analysis revealed that positively charged residues are widely distributed across diverse DNA-binding regions and typically exhibit high evolutionary conservation. Fig. 5 shows the sequence logos of six representative DNA-binding regions from Rel, zinc finger, homeobox, basic region leucine zipper, serum response factor, and helix-loop-helix transcription factors.

## Discussion

The velvet family of transcriptional and epigenetic regulatory proteins represents an ancient class of DNA-binding proteins, with orthologs traceable to the pre-fungal ancestor that predated the divergence of the opisthokont lineage (Chen et al. 2024). Velvet regulators have attracted broad attention from fungal scientists due to their wide distribution in the fungal kingdom and their unique and crucial roles in fungal development, secondary metabolism, stress response, and pathogenicity (Chen et al. 2026). Recently, the general architecture of velvet domains was proposed by dividing them into an approximately 30-amino-acid N-terminal DNA-binding region and a roughly 100-amino-acid C-terminal dimerization region, which markedly clarifies the working mechanisms of velvet proteins (Chen et al. 2025).

In the present work, the DNA-binding region of velvet proteins was systemically characterized using *A. nidulans* VelB as a paradigm. Through alanine-scanning mutagenesis targeting 15 conserved amino acid residues within this DNA-binding region of *A. nidulans* VelB, three key positively charged residues, namely R71, R80, and R81, were identified (Fig. 1). Subsequent functional assays demonstrated that these three residues are essential for the full function of VelB, including the maintenance of long-term spore viability, regulation of sexual development, and modulation of secondary metabolism (Fig. 2). Structural analysis of the VosA homodimer and VosA–VelB heterodimer revealed a strongly electropositive surface across the velvet DNA-binding region, and the three corresponding positively charged residues in VosA were verified to have important roles in DNA binding (Ahmed et al. 2013). To clarify the effects of the above mutations on surface electrostatic potential, the electrostatic profiles of wild-type VelB and its mutant variants were compared. The results showed that individual single-point mutations not only significantly lower local electropositive potential but also provoke long-range electrostatic perturbations, ultimately altering the global surface profile critical to molecular recognition and protein functionality (Fig. 3; Table 2).

These three positions, which are invariably occupied by positively charged basic residues, exhibit strict evolutionary conservation across velvet domains from all major taxonomic groups. Frequency analysis of 4,999 non-redundant velvet domain sequences revealed that position 71 is occupied by R in 85% of sequences and K in 4%; position 80 by R in 91% and K in 2%; and position 81 by R in 84% and K in 9% (Fig. 1A). To test the functional conservation of the velvet DNA-binding domain, a cross-kingdom domain-swapping experiment was performed, in which the endogenous DNA-binding region of *A. nidulans* VelB was replaced with the orthologous region from *C. owczarzaki*—a unicellular opisthokont that diverged prior to the emergence of the fungal kingdom (Fig. 4). However, expression of this chimeric VelB construct in a *velB* deletion strain failed to rescue the full spectrum of *velB* mutant phenotypes, with only partial restoration of sexual development observed under dark conditions. Sequence alignment revealed that the *Capsaspora* velvet domain specifically lacks the critical arginine residue at the position corresponding to R71 in VelB (Fig. 4A), which is essential for VelB function (Fig. 2). To directly test whether this single residue difference accounts for the functional incompatibility, an arginine residue at the corresponding position in the *Capsaspora* velvet DNA-binding region was artificially introduced to generate the modified chimeric strain *velB (∆velB_DBR::CapVelvet_DBR)^R71^*. Strikingly, expression of this modified chimeric protein in the *velB* deletion strain was sufficient to substantially rescue all tested mutant phenotypes, including long-term spore viability, sexual development, and colony pigmentation (Fig. 4B, D, E). These findings strongly suggest that while the high sequence conservation of the velvet DNA-binding domain underpins a broadly conserved core mechanism of protein–DNA interaction, subtle functional divergence may have emerged among distinct velvet family members.

In summary, using *A. nidulans* VelB as a paradigm, the present study identified three critical positively charged arginine residues within the velvet DNA-binding region that are indispensable for VelB function. This essential conserved arginine cluster constitutes the structural prerequisites for generating the strongly electropositive surface potential that mediates specific interactions between velvet proteins and DNA. These findings not only advance the mechanistic understanding of fungal velvet regulators but also provide general insights into the molecular basis of DNA recognition. Indeed, surface-exposed positively charged residues represent a universal feature of diverse DNA-binding motifs, including those of the Rel homology domain, basic region leucine zipper, and helix-loop-helix transcription factors (Fig. 5).

## Additional information

### Artificial Intelligence (AI) use

The authors accept full responsibility for the content of the manuscript, including the disclosure of any use of AI.

No AI tools were used in the preparation of this manuscript.

### Adherence to national and international regulations

All the fungal strains used in this study have been legally obtained, respecting the Convention on Biological Diversity (Rio Convention).

### Author ORCIDs

W. Chen https://orcid.org/0000-0002-6849-1972

A.M. Köhler https://orcid.org/0000-0002-2715-8954

G.H. Braus https://orcid.org/0000-0002-3117-5626

### Data availability

All of the data that support the findings of this study are available in the main text or Supplementary Information.

## Supplementary materials

Supplementary material 1 Supplementary tables Data type pdf Explanation note **table SS1**. Plasmids constructed and used in this study. natRM = nourseothricin recyclable marker cassette, phleoRM = phleomycin recyclable marker cassette. **table SS2**. Oligonucleotides utilized in this study. https://binary.pensoft.net/file/1715382This dataset is made available under the Open Database License (http://opendatacommons.org/licenses/odbl/1.0/). The Open Database License (ODbL) is a license agreement intended to allow users to freely share, modify, and use this Dataset while maintaining this same freedom for others, provided that the original source and author(s) are credited. Wanping Chen, Anna M. Köhler, Mingrong Deng, Honghui Zhu, Gerhard H. Braus

Supplementary material 2 Modeled structure files in CIF format Data type rar Explanation note Supplemental data–the modeled structure files of VelB and the R71A, R80A, and R81A variants in CIF format. https://binary.pensoft.net/file/1715383This dataset is made available under the Open Database License (http://opendatacommons.org/licenses/odbl/1.0/). The Open Database License (ODbL) is a license agreement intended to allow users to freely share, modify, and use this Dataset while maintaining this same freedom for others, provided that the original source and author(s) are credited. Wanping Chen, Anna M. Köhler, Mingrong Deng, Honghui Zhu, Gerhard H. Braus
