# Supplementary material for: Functional dissection of velvet DNA-binding region in Aspergillus nidulans VelB uncovers an essential conserved arginine cluster
Source: IMA Fungus. 2026 Jul 14;17:e204179. doi: 10.3897/imafungus.17.204179 (PMC13389560; doi:10.3897/imafungus.17.204179)
Supplement: Supplementary material 1 — Supplementary tables [file imafungus-17-e204179-s001.pdf]

Materials and Methods

Plasmid construction and preparation

For plasmid construction, target DNA fragments were amplified by polymerase chain reaction (PCR). The amplified fragments were ligated into the designated restriction sites of plasmids using the Seamless Cloning and Assembly Kit (Invitrogen). Plasmids were then propagated in *E. coli* and extracted using the Qiaprep Spin Miniprep Kit (Qiagen) in accordance with the manufacturer's instructions. All plasmids generated and employed in this work are listed in Table S1.

Table S1. Plasmids constructed and used in this study. natRM = nourseothricin recyclable marker cassette, phleoRM = phleomycin recyclable marker cassette

Plasmid	Description	Source
pME4319	The recyclable marker cassette-containing plasmid with the bleo gene conferring resistance to phleomycin	(Liu et al. 2021)
pME4687	*A. nidulans velB* with sgfp tag: velB::sgfp::natRM	(Kohler et al. 2026)
pME5332	*A. nidulans velB* 3UTR integrated in the PmlI restriction cutting site of plasmid pME4319	(Chen et al. 2025)
pME5333	*A. nidulans* velB_5UTR:velBcDNA: sgfp: phleoRM:velB_3UTR	(Chen et al. 2025)
pME5335	*A. nidulans* velB_5UTR: velBcDNA (ΔvelB_DBR::CapVelvet_DBR): sgfp: phleoRM: velB_3UTR	This study
pME5338	*A. nidulans* velB_5UTR: velB^{L61A}:sgfp: phleoRM: velB_3UTR	This study
pME5339	*A. nidulans* velB_5UTR: velB^{Q65A}:sgfp: phleoRM: velB_3UTR	This study
pME5340	*A. nidulans* velB_5UTR: velB^{P67A}:sgfp: phleoRM: velB_3UTR	This study
pME5341	*A. nidulans* velB_5UTR: velB^{R71A}:sgfp: phleoRM: velB_3UTR	This study
pME5342	*A. nidulans* velB_5UTR: velB^{C73A}:sgfp: phleoRM: velB_3UTR	This study
pME5343	*A. nidulans* velB_5UTR: velB^{G74A}:sgfp: phleoRM: velB_3UTR	This study
pME5344	*A. nidulans* velB_5UTR: velB^{G76A}:sgfp: phleoRM: velB_3UTR	This study
pME5345	*A. nidulans* velB_5UTR: velB^{K78A}:sgfp: phleoRM: velB_3UTR	This study
pME5346	*A. nidulans* velB_5UTR: velB^{D79A}:sgfp:	This study

	phleoRM: velB_3UTR	
pME5347	*A. nidulans* velB_5UTR: velB^{R80A}:sgfp: phleoRM: velB_3UTR	This study
pME5348	*A. nidulans* velB_5UTR: velB^{R81A}:sgfp: phleoRM: velB_3UTR	This study
pME5349	*A. nidulans* velB_5UTR: velB^{P82A}:sgfp: phleoRM: velB_3UTR	This study
pME5350	*A. nidulans* velB_5UTR: velB^{P85A}:sgfp: phleoRM: velB_3UTR	This study
pME5351	*A. nidulans* velB_5UTR: velB^{P86A}:sgfp: phleoRM: velB_3UTR	This study
pME5352	*A. nidulans* velB_5UTR: velB^{P87A}:sgfp: phleoRM: velB_3UTR	This study
pME5353	*A. nidulans* velB_5UTR: velBcDNA (ΔvelB_DBR:: CapVelvet_DBR)^{R71}:sgfp: phleoRM: velB_3UTR	This study

Oligonucleotides used in this study

Oligonucleotides used in this study are listed in Table S2.

Table S2. Oligonucleotides utilized in this study

Oligo Name	Sequence (5’-3’)
WC12	ATAGGCCTGAGATTTCTACTTGTACAGTTCGTCCAT
WC30	TGCAGGAATTCGATATTTGTTTAAAC
WC154	ACACCACCCATGCAGGCGTGCTTGGGCTGCTGGCGGATGTG CAAGGACCAGATACGACC
WC155	CCTGCATGGGTGGTGTCTCCCACGACGGTGCCCGCGGTAAC CGAAGGCCAATCACACCC
WC171	CACGACTTGCAAGGACCAGAT
WC172	GTCCTTGCAAGTCGTGGCACAGCCGATTCGCGC
WC173	CTGTTGCACGACTTGCAAGG
WC174	GCAAGTCGTGCAACAGGCGATTCGCGCGCGGA
WC175	CGCGCGAATCGGCTGTT
WC176	ACAGCCGATTCGCGCGGCGATGTGCGGCTTTGG
WC177	CGCACATCCGCGCGCGAAT
WC178	CGCGCGCGGATGTGCGCCTTTGGCGATAAGGT
WC179	ATCGCCAAAGCCGCACATC
WC180	GTGCGGCTTTGGCGATGCGGTTGGCTATTTGTA
WC181	CCTAGAGAGAGTCAGCACGGAC
WC182	GCTGACTCTCTCTAGGCCCGAAGGCCAATCAC
WC183	GTCCTAGAGAGAGTCAGCACGG
WC184	TGACTCTCTCTAGGACGCAAGGCCAATCACACC

WC185	TCGGTCCTAGAGAGAGTCAGCA
WC186	CTCTCTCTAGGACCGAGCGCCAATCACACCCCC
WC187	CCTTCGGTCCTAGAGAGAGTCA
WC188	TCTCTAGGACCGAAGGGCAATCACACCCCCTC
WC189	TGTGATTGGCCTTCGGTCCT
WC190	CCGAAGGCCAATCACAGCCCCTCCGTGTATCC
WC191	GGGTGTGATTGGCCTTCGGT
WC192	AAGGCCAATCACACCCGCTCCGTGTATCCGTC
WC193	AGGGGGTGTGATTGGCCTT
WC194	GCCAATCACACCCCCTGCGTGTATCCGTCTAA
WC208	GGACCAGATACGACCGTCATG
WC209	CGGTCGTATCTGGTCCGCGCAAGTCGTGCAACA
WC210	CAAAGCCGCACATCCGCGCG
WC211	CGGATGTGCGGCTTTGCCGATAAGGTTGGCTA
WC212	CATGCAACGGGCGTGCTTGGGCTGCTGGC
WC213	GCACGCCCGTTGCATGGGTGGTGTCTCCCA
WC251	CATCCGCGCGCGAATCGGCTGTT
WC252	GATTCGCGCGCGGATGGCCGGCTTTGGCGATAA

Note: the underlined oligonucleotides were generated the overlaps for seamless cloning in plasmid construction.

References

Chen W, Sasse C, Köhler AM, Wang D, Popova B, Strohdiek A, Braus GH (2025) Resolving the fungal velvet domain architecture by *Aspergillus nidulans* VelB. mBio 16: e00261-00225. https:// doi:10.1128/mbio.00261-25.

Kohler AM, Thieme S, Gerke J, Thieme KG, Harting R, Schmitt K, Valerius O, Chen W, Hofer A, Bastakis E, et al. (2026) The VelB IDD promotes selective heterodimer formation of velvet proteins for fungal development. Life Sci Alliance 9: e202503395. https:// 10.26508/lsa.202503395.

Liu L, Sasse C, Dirnberger B, Valerius O, Fekete-Szucs E, Harting R, Nordzieke DE, Poggeler S, Karlovsky P, Gerke J, et al. (2021) Secondary metabolites of Hülle cells mediate protection of fungal reproductive and overwintering structures against fungivorous animals. eLife 10: e68058. https:// 10.7554/eLife.68058.
